# Larval Zebrafish Model for FDA-Approved Drug Repositioning for Tobacco Dependence Treatment

**DOI:** 10.1371/journal.pone.0090467

**Published:** 2014-03-21

**Authors:** Margot A. Cousin, Jon O. Ebbert, Amanda R. Wiinamaki, Mark D. Urban, David P. Argue, Stephen C. Ekker, Eric W. Klee

**Affiliations:** 1 Mayo Addiction Research Center, Mayo Clinic, Rochester, Minnesota, United States of America; 2 Center for Clinical and Translational Science, Mayo Clinic, Rochester, Minnesota, United States of America; 3 Nicotine Dependence Center, Mayo Clinic, Rochester, Minnesota, United States of America; Tulane University Medical School, United States of America

## Abstract

Cigarette smoking remains the most preventable cause of death and excess health care costs in the United States, and is a leading cause of death among alcoholics. Long-term tobacco abstinence rates are low, and pharmacotherapeutic options are limited. Repositioning medications approved by the U.S. Food and Drug Administration (FDA) may efficiently provide clinicians with new treatment options. We developed a drug-repositioning paradigm using larval zebrafish locomotion and established predictive clinical validity using FDA-approved smoking cessation therapeutics. We evaluated 39 physician-vetted medications for nicotine-induced locomotor activation blockade. We further evaluated candidate medications for altered ethanol response, as well as in combination with varenicline for nicotine-response attenuation. Six medications specifically inhibited the nicotine response. Among this set, apomorphine and topiramate blocked both nicotine and ethanol responses. Both positively interact with varenicline in the Bliss Independence test, indicating potential synergistic interactions suggesting these are candidates for translation into Phase II clinical trials for smoking cessation.

## Introduction

Despite ongoing public health efforts and treatment advances, cigarette smoking rates in the United States have not decreased over the last decade [1]. Two-thirds of cigarette smokers have attempted to quit at least once and one-half try to quit every year [2]; however, only seven percent achieve tobacco abstinence at one year [3]. Additionally, over 70% of alcoholics smoke, and tobacco-related disease is a leading cause of death among patients with alcohol use disorder [4]. Pharmacotherapy is a cornerstone in tobacco dependence treatment, but not all smokers achieve abstinence with the current medications, and relapse rates remain high. Novel pharmacotherapies are needed, and those maintaining efficacy in treating alcohol-dependent smokers would have high utility for this subset of smokers.

Substance use disorders are complex with molecular, genetic, and social correlates impacting abstinence. Substance use disorders are associated with a spectrum of endophenotypes [5], [6] as well as behavior patterns and symptoms such as drug seeking, impulsivity, and withdrawal. Some aspects of drug use are manifested only in the context of a substance use disorder (i.e., compulsivity and impulsivity), while others are responses to the drug itself (i.e., reward) and not a specific phenotype of a substance use disorder. Many of these behaviors can be modeled in animals including zebrafish [7]. Although not representative of all aspects of a substance use disorder, locomotor activation is a component behavior that models the unconditioned response to the rewarding nature of drugs of abuse following a single drug exposure. Acute drug exposure leads to dopamine release and increased activity [8] providing a direct readout of sensitivity to the behavioral effects of nicotine and ethanol, a feature that, in humans, is linked to the propensity to use these drugs.

An animal model with predictive clinical validity offers an efficient and cost-effective strategy for repositioning medications approved by the FDA. Medication repositioning provides an opportunity to add novel pharmacotherapeutics to the tobacco dependence treatment armamentarium while circumventing the enormous investment associated with new drug development [9], [10]. Zebrafish are a vertebrate model system amenable to the study of substance use disorders [11]–[13] and are increasingly used for *in vivo* drug-repositioning [14]–[16] studies.

We hypothesized that the modulation of nicotine-induced locomotion in zebrafish could predict clinical efficacy of novel medications for the treatment of tobacco dependence. We developed an assay to identify candidate medications and established predictive clinical validity of this assay with current front-line therapeutics for tobacco dependence treatment, such as varenicline and bupropion. We then evaluated a physician-vetted panel of FDA-approved medications amenable to rapid translation into clinical evaluation in humans. We further studied the effects of candidate medications on ethanol-induced locomotor activation not only to better characterize specificity for nicotine response modulation, but also to identify those medications more likely to aid the smoker with comorbid alcohol use disorder.

## Materials and Methods

### Ethics Statement

Zebrafish larvae were raised within the Mayo Clinic Zebrafish Core Facility with adherence to the NIH Guide for the Care and Use of Laboratory Animals and approval by Mayo Clinic's Institutional Animal Care and Use Committee (A21710).

### Zebrafish

Wild-type zebrafish (*Danio rerio*) were purchased from Segrest Farms and bred within the Mayo Clinic Zebrafish Core Facility. Embryos and larvae were maintained in 0.5X E2 media (embryo water) without methylene blue as described by the Zebrafish International Resource Center [17]. At day 0, embryos were collected and transferred into groups of 60 per 100-mm petri dish. Day 1, the dead/non-viable embryos were removed. All embryos were kept at 28°C on a 14/10-hour light and dark schedule. At 3–4 days post fertilization (dpf), larvae were transferred into groups of 10 in 35-mm Petri dishes. At 5dpf, larvae were pre-treated in the test compound(s) or an equivalent volume of embryo water and incubated overnight. The following morning, the larvae were transferred to small (41×41×8 mm) weighing dishes (Fisher, cat. # 08-732-112) for testing.

### Nicotine Locomotor Activation Assay

This assay is an extension of a previously published nicotine-activated locomotor response assay in larval zebrafish [18], utilizing video capture and a total distance moved locomotion metric. The refined method automates data analysis, calculating per-second cumulative distance moved for 10–15 larval zebrafish per test condition, run in triplicate. For each test, larvae were acclimated to the testing light-box apparatus for 20 minutes prior to testing. The experimental setup is shown in **Figure S1**. Two minutes of baseline activity is captured after which 500 μl of either 400 μM nicotine (Acros Organics, cat. # 181420050) or embryo water is added to the fish water making a total volume of 10 ml per dish (for a final nicotine concentration of 20 μM). The baseline activity and five minutes of post-stimulus exposure are captured on video and analyzed at one frame-per-second.

### Drugs and Dosing

The compounds and vehicles used in this study to test for modulation of the nicotine-induced locomotor activation are described in **Table S1**. Those medications obtained in tablet formulation were crushed with a mortar and pestle before suspending in DMSO or embryo water. For all drug pretreatment experiments, fish were administered the drug in the fish water at 5 dpf in the afternoon and challenged with nicotine the following day (6 dpf). Mecamylamine was tested at 10 μM consistent with previous studies [18] and hexamethonium was tested at 5,000 μM. The high dose of hexamethonium was used to eliminate the chance of sub threshold dosing. Lesser concentrations of hexamethonium in bath application have been shown to elicit appropriate responses in fish *in vivo* [19], [20]. The remaining compounds were initially tested at doses of 10 and 50 μM. If no effect was measured, toxicity testing from 75 μM to 1 mM was performed in which the fish were incubated in the drug overnight. If a phenotype (death, sluggish swimming, failure to respond to a startle, etc.) was observed, a dose halfway between the effect-inducing dose and the next lower dose tested was selected for further evaluation. Similarly, if the 10 and 50 μM concentrations were lethal, we performed toxicity testing at lower doses to determine an appropriate starting dose. Finally, if attenuation of the nicotine-induced locomotor activation is seen, the dose is titrated to obtain at least 30% of the response of the non-pretreated fish to nicotine and achieve statistical significance. This dose is then tested with the control stimuli, cinnamon oil and mustard oil, to determine if the reduced nicotine response is due to peripheral or sedating effects of the medications.

### Ethanol Locomotor Activation Assay

The assay is performed as described in the nicotine locomotor activation assay, except the recording of the larval locomotion occurred from 30 to 40 minutes after administering ethanol (Sigma, cat. # E7023) or embryo water into the larval water. Final concentrations of ethanol for the dose-response experiment included 0%, 0.5%, 1%, 2% and 4% and were run in triplicate. All counter screening experiments of pharmacotherapeutics used a final concentration of 1.5% ethanol in the water of the larvae, with six replicates of 10 larvae for each condition, and the same medication dose we reported on for the nicotine assay. The additional replicates used here, not used in the nicotine assays, were required to attain the power to detect pharmacotherapeutic modulation of this ethanol response, which increases larval locomotion to a lesser magnitude than nicotine.

### Locomotor Activation Controls

Two non-nicotine stimuli, cinnamon oil (Sigma, cat# C7267, 25 ml) and mustard oil (Sigma, cat#377430, 5 g), were used to assess if the drug-induced attenuation of the nicotine response was due to impairment of overall swimming ability or response (sedation or paralysis, for example). These controls were tested independently using an identical protocol to the nicotine-induced locomotor activation studies, with substitution of either cinnamon oil (50 μM) or mustard oil (25 μM) [21] for nicotine as the stimulating agent.

A third control assay was performed to ascertain the potential for irreversible effects or damage caused by exposure to the testing compound. For this assay, the fish were tested using the normal nicotine-induced locomotor activation protocol, but following the assay the zebrafish larvae were removed from the nicotine and drug solution, thoroughly rinsed, replaced in clean embryo water for 24 hours, and then retested for nicotine-induced locomotor activation.

Lastly, an evaluation of the acute effects of those medications attenuating the nicotine locomotor response, but not cinnamon oil or mustard oil, on larval zebrafish locomotion was conducted. Larval fish at 5 dpf in the afternoon were challenged with the medication or an equivalent volume of embryo water in the locomotor assay. The assay design is comparable to that of the nicotine assay except the medication is used as the stimulus and the fish are recorded for 30 minutes after administration of the medication. For comparison, a nicotine control was also run in parallel with the medication and water control groups. Data were quantified for the first four minutes and the 26 to 30 minute post administration timeframes as the total distance traveled as a percent of nicotine response.

### Image analysis software

Video analysis is performed using a software program developed using MATLAB Version 7.11.0.584 (R2010b). The program utilizes components of the MATLAB Image Processing Toolbox (version 7.11) add-on package to perform specialized high-throughput analysis of zebrafish larvae behavioral video frames. The graphical user interface (GUI) was designed using MATLAB's GUI Design Environment (GUIDE) and offers numerous configuration options, including accommodations for baseline measurements, periodic sampling, different sized weighing dishes, alternate movie resolutions, different plate configurations and batch processing of multiple input videos. A debug mode allows the user to visually verify that the software is correctly identifying the areas of interest within each video frame. Result data showing various metrics (number of fish moved, distance moved, pixel count) are outputted to comma-separate value (CSV) files, which can be further analyzed in other programs. The software is compiled to run as a standalone program utilizing the MATLAB Compiler Runtime (MCR) program, which enables the execution of MATLAB programs without a full MATLAB installation and license.

Weighing dishes (Fisher Sci., cat. # 08-732-112) are used in our experiments because of the low cost, good transparency, and angled sides. The angled sides keep the larvae in clear view of the camera and prevent reflection issues with the dish sides that hinder the accurate measurement of larval movements when using petri dishes. Multiple larvae can be placed within the same weighing dish increasing the number of fish that can be screened at one time. The weighing dishes are placed on a LED plate with a 1/8-inch thick sheet of white acrylic in between to diffuse the light evenly (**Fig. S1**). When using a Whitegoods LightMeter app (whitegoods.com) on an iphone 5, luminance was measured from the top of the weighing dish through the weighing dish and white acrylic sheet and found to be approximately 1.2 klux.

Custom clear acrylic templates hold the weighing dishes in the proper location during filming. The software uses circular alignment dots on the template to align each movie frame and adjust for variable zoom levels between assays. Once aligned, each weighing dish location is determined relative to the position of the left-most alignment dot. Then, each frame is opened sequentially, analyzed against the comparison frame, and closed, so the maximum movie file size that can be analyzed is not restricted by available memory. Each frame is subtracted from the comparison frame to determine pixel differences, indicating larval movement, between the two frames. The center of each contiguous collection of pixels meeting minimum threshold requirements is used as the point of detected larval movement. A closest point algorithm is used to match the before and after locations of multiple zebrafish larvae in the same dish. Drug (or placebo) administration is indicated in the movie by placing a penny in the frame, which is detected by the software and flagged in the output results. After locating the penny in the movie, movement analysis takes approximately one second for each desired frame comparison when the software is run on a 2.66 GHz Intel Core 2 Duo iMac computer.

Software accuracy was tested by comparing the software output CSV files to manual comparisons of 591 larval movements across 60 movie frames with two weighing dishes, each holding 10 larvae. The software accurately detected 98.6% of movements and correctly matched the before and after location of the larvae in 97.3% of cases. The majority of errors occurred when a larva twisted, rather than swam forward or backward, resulting in two detected movements instead of a just one.

### Statistical Analysis

The results were summarized as the total distance traveled per second over time for each condition, averaged across replicates, and taken as a percentage of the stimulus-only response. The average cumulative distances traveled for 0–4 minutes post nicotine exposure was calculated for each condition. A two-sided t-test (alpha = 0.05) was then used to assess significance when comparing the drug pretreatment to stimulus-only response.

### Combination therapy and drug interaction

Apomorphine, bupropion, betaxolol, carisoprodol, clonazepam, diazepam, lorazepam, topiramate, and zolpidem were all assessed for interaction with varenicline in a combination treatment experiment. The nicotine-induced locomotor activity assay was performed as described above with six replicates of the following conditions: drug pretreatment + nicotine, varenicline pretreatment + nicotine, drug + varenicline pretreatment + nicotine, no pretreatment + nicotine. Doses used for this experiment were at half the concentration found to be effective for attenuation of the nicotine response. The data were analyzed using a Bliss Independence model comparing the expected response of combination therapy to the measured experimental response. The expected percent nicotine response (E) was calculated using E = 1−(D+V−D×V), where D =  % attenuation of nicotine response in drug treated larvae and V =  % attenuation of nicotine response in varenicline treated larvae. We then plotted the expected percent nicotine response against the measured response.

## Results

### Nicotine-Induced Locomotor Activity

**Figure 1:**
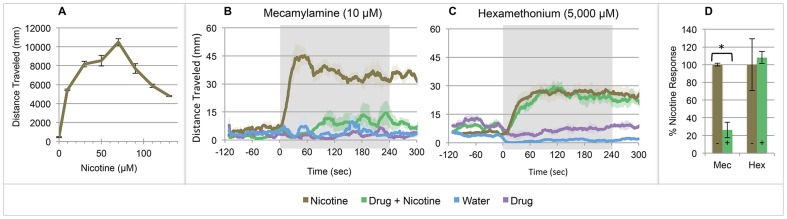
Larval zebrafish respond to nicotine. Increased locomotion occurs immediately following full-body exposure in 20 μM nicotine. (**A**) The inverted-U dose response. (**B–C**) Pretreatment with nAChR antagonists with nicotine challenge (± SE): (**B**) mecamylamine (10 μM), (**C**) hexamethonium (5,000 μM, p = 0.803), a peripheral nervous system-only nAChR antagonist. (**D**) Mean cumulative distance traveled in the first 4 minutes post-nicotine exposure as a percent of the average untreated nicotine response (± SE). n≥30 larvae per condition; *  = p<0.05; Students t-test.

Acute nicotine exposure at doses from 10 to 130 μM rapidly induces a locomotor response in larval zebrafish that recapitulates the inverted-U response (**Fig. 1A**) described in other preclinical models [18], [22]–[25]. We selected a 20 μM dose of nicotine to evaluate the pharmacotherapeutic modulation of this response. To establish central nervous system (CNS) contribution to this nicotine-induced locomotor activation, larvae were pretreated with the non-specific nicotinic acetylcholine receptor (nAChR) antagonists, mecamylamine or hexamethonium, prior to experimentation. Mecamylamine blocked the nicotine response (**Fig. 1B,D**) consistent with Petzold *et al* [18], but hexamethonium, which fails to cross the blood-brain barrier, did not (**Fig. 1C,D**), suggesting CNS nAChR activation is required for nicotine-induced locomotion.

### Pharmacotherapeutic Attenuation of Nicotine-Induced Locomotor Activity

**Figure 2:**
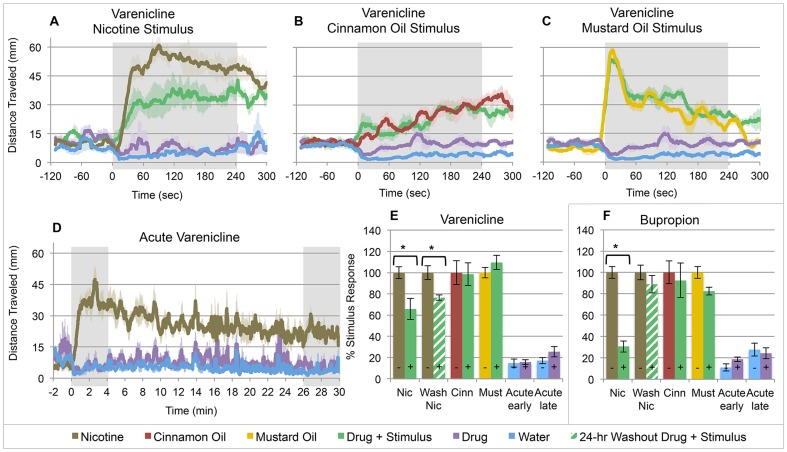
Tobacco dependence treatment medications (varenicline, bupropion) alter the larval zebrafish nicotine response. (**A–C**) Larvae pretreated in varenicline (50 μM) overnight and challenged with stimulus at 6 dpf (± SE). Varenicline attenuates (**A**) 20 μM nicotine response, but not (**B**) 50 μM cinnamon oil or (**C**) 25 μM mustard oil response. (**D**) Acute treatment with 50 μM varenicline does not affect locomotion at 5 dpf (± SE). (**E–F**) Mean cumulative distance traveled in the 4 minutes post stimulus exposure as a percent of the average untreated stimulus response (± SE). Wash Nic = 24-hour washout period following acute nicotine experiment and re-tested at 7 dpf. Acute early and acute late response represents the first 4 minutes and last 4 minutes, respectively, post drug exposure at 5 dpf. (**E**) Movement quantitation for varenicline experiments. (**F**) Movement quantitation for bupropion experiments. n≥30 larvae per condition; *  = p<0.05; Students t-test.

Larval zebrafish were pretreated with each of the two FDA-approved smoking cessation medications to establish predictive clinical validity. Overnight pretreatment with 50 μM varenicline or 10 μM bupropion exhibited continuously attenuated locomotor activation following exposure to 20 μM nicotine, compared to untreated controls, without impacting baseline activity (**Fig. 2A,E,F**). This is consistent with the clinical efficacy of varenicline and bupropion for smoking cessation. To eliminate sedation or swimming impairment as the cause of locomotor attenuation, cinnamon oil and mustard oil were used in place of nicotine in the locomotor activation assay. These chemicals have previously been shown to increase larval zebrafish locomotion through a peripheral sensory neuron response [21]. We observed no significant attenuation of either cinnamon oil- or mustard oil-induced locomotion following varenicline or bupropion pretreatment (**Fig. 2B,C,E,F**). Acute exposure to varenicline (**Fig. 2D,E**) or bupropion (**Fig. 2F**) also did not alter locomotion. Additionally, 24-hour removal from varenicline exposure resulted in partial recovery (**Fig. 2E**), and bupropion exposure resulted in full recovery (**Fig. 2F**) of the nicotine response. The varenicline-treated fish in the recovery experiments behaved normally by inspection of swimming behavior and were otherwise healthy. The incomplete recovery of nascent nicotine-induced activity in the varenicline-treated larvae may result from the clearance or binding affinity of varenicline in zebrafish that has yet to be studied. In mammalian studies, varenicline was cleared renally by active and passive mechanisms in mostly unchanged active form and has a half-life of 24 hours [26], [27].

### Novel Therapeutic Evaluation

**Figure 3:**
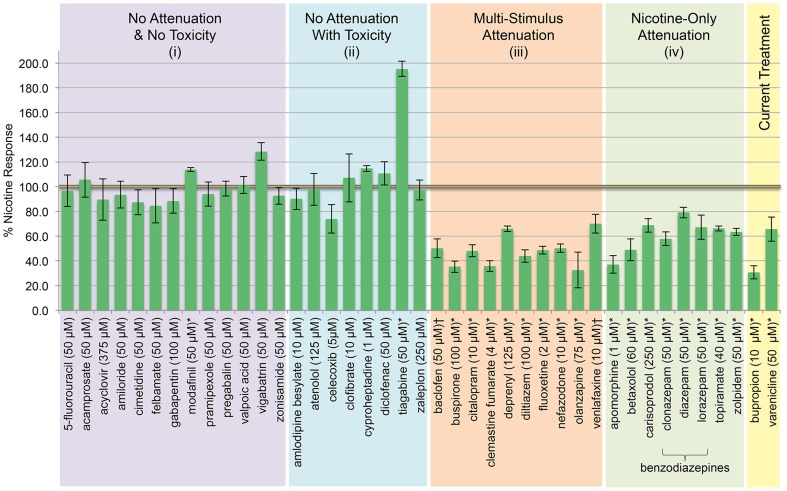
Evaluating FDA-approved medications identifies new modifiers of the nicotine response using larval zebrafish locomotion. Zebrafish pretreated with medication overnight and challenged with 20 μM nicotine. Category (i) compounds: no attenuation of the nicotine response and no toxicity with overnight incubation in the drug up to 1 mM concentrations. Category (ii) compounds: no attenuation of the nicotine response, but toxicity was observed at the next highest dose evaluated. Category (iii) compounds: significantly attenuated the nicotine response and the cinnamon oil and/or mustard oil response. Category (iv) compounds: significantly attenuated the locomotor response to nicotine, but not to cinnamon oil or mustard oil responses. Current Treatment  =  FDA-approved medications for smoking cessation. n≥30 larvae per condition; *  = p<0.05; † = 0.05<p<0.1; Students t-test.

We tested 39 additional FDA-approved medications for nicotine response blockade (**Table S1**). Results are in **Figure 3** and **Table S2** and can be summarized as: (i) no attenuation with no toxicity, (ii) no attenuation, (iii) multi-stimulus attenuation (cinnamon oil and/or mustard oil in addition to nicotine), and (iv) nicotine-only attenuation (normal response to locomotor controls). Thirteen category (i) pharmacotherapeutics failed to attenuate nicotine locomotor activation and induced no phenotypic effects during toxicity testing. Eight category (ii) medications failed to attenuate the nicotine response, but showed a phenotype during the toxicity testing suggesting drug absorption. Ten category (iii) medications significantly attenuated the nicotine, and cinnamon oil and/or mustard oil locomotor activation. Eight category (iv) compounds elicited statistically significant attenuation of the nicotine response, but not control stimuli. These include apomorphine, betaxolol, carisoprodol, clonazepam, diazepam, lorazepam, topiramate, and zolpidem. DMSO (1%) vehicle control had no effect on locomotor response to any stimulus tested in this study (**Table S2**), and was tested at a higher concentration than used to reconstitute any of the evaluated medications. Vehicle concentrations are informed in **Table S1**.

### Ethanol Evaluation

**Figure 4:**
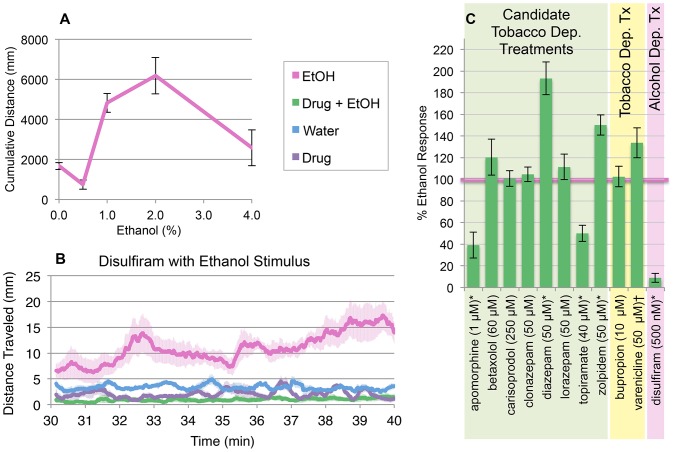
Nicotine-modulating FDA-approved drugs show distinct effects on ethanol-induced locomotor activation. (**A**) Inverted-U dose response on locomotion from 0 to 4% ethanol. Cumulative distance is the mean of the summed one-second distances 30–40 minutes post bath ethanol exposure (± SE); n = 30 larvae per condition. (**B**) Ethanol (1.5%)-induced locomotion is decreased with overnight pretreatment in disulfiram (500 nM); n = 60 larvae per condition (± SE). (**C**) Larvae pretreated overnight in medication are subsequently challenged with 1.5% ethanol. Bars represent mean cumulative distance traveled during the 30–40 minutes post-ethanol exposure as a percent of the average untreated ethanol response (± SE); n = 60 larvae per condition; *  = p<0.05; † = 0.05<p<0.1; students t-test.

Locomotor activation following ethanol exposure [28] was also evaluated for the category (ii) and (iv) medications to further characterize the specificity of the nicotine-response modifiers and to identify medications attenuating the effects of both nicotine and ethanol. Ethanol exposure increases larval locomotion (30–40-minute time interval) post administration compared to controls. An inverted-U shaped dose-response curve was observed (**Fig. 4A**) and a 1.5% ethanol concentration was used for all drug evaluations. Predictive clinical utility was established with disulfiram, an ethanol metabolism inhibitor used to treat chronic alcoholism. Disulfiram (500 nM) attenuated the ethanol response, consistent with the clinical efficacy of this medication (**Fig. 4B,C**), and failed to affect the nicotine response at this dose (**Table S2**). Two category (iv) compounds, apomorphine and topiramate, attenuated both nicotine and ethanol responses (**Fig. 4C**).

### Combination Pharmacotherapy

**Figure 5:**
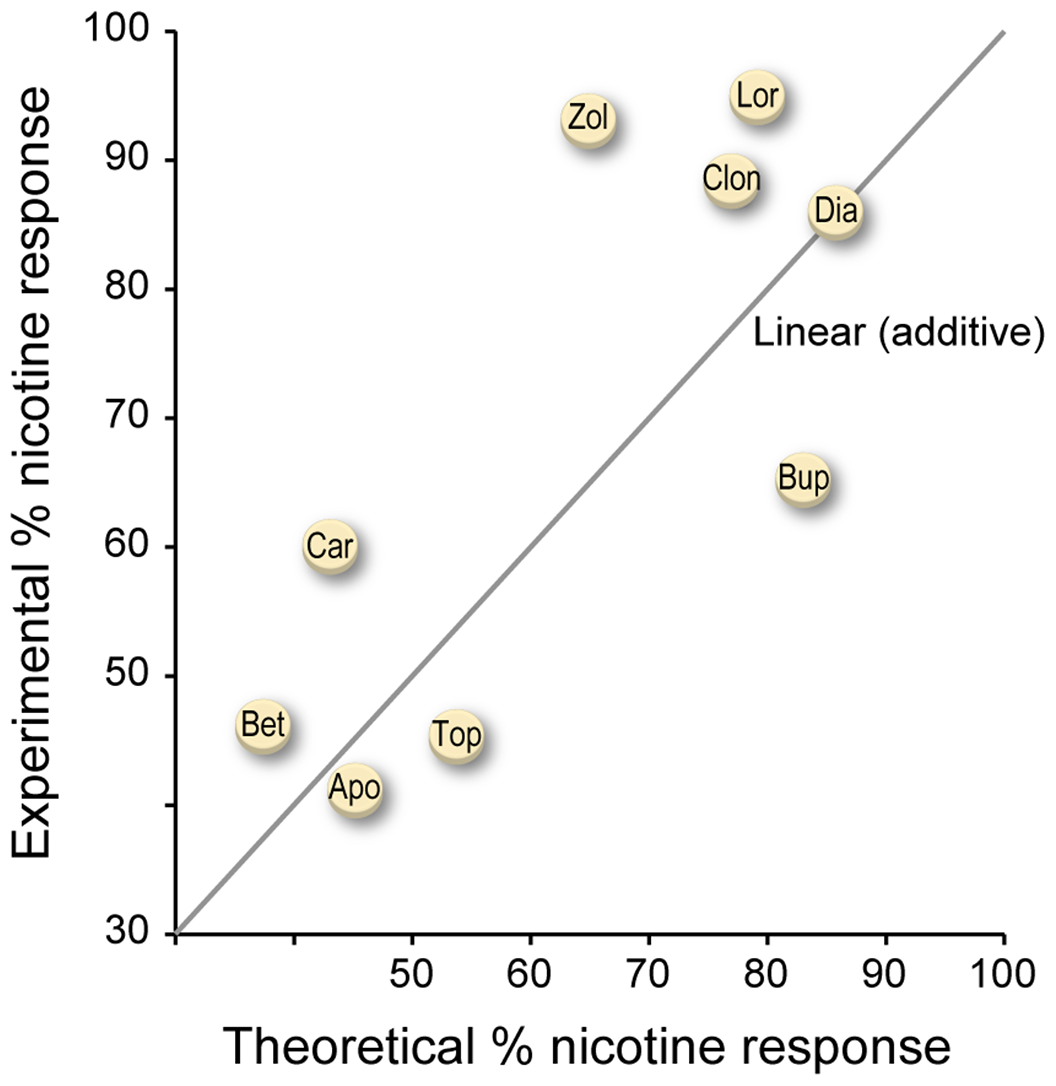
Combination therapy assessment with varenicline. One known and two new potential synergistic interactions with varenicline have been identified. Bupropion and topiramate show a positive interaction with varenicline using Bliss Independence analysis. Larval zebrafish were pretreated in each medication and varenicline at 50% of the monotherapy dose found to be effective. Six replicates of 10 larvae per condition were challenged with 20 μM nicotine. Conditions included drug pretreated, varenicline pretreated, drug and varenicline pretreated and untreated larvae. We calculated the expected effect of each combination with the equation: E = D×V, where D =  % nicotine response of drug treated larvae and V =  % nicotine response of varenicline treated larvae. The experimental percent nicotine response is plotted against the theoretical calculated response. Apomorphine, bupropion, and topiramate show a greater-than-additive effect, diazepam and betaxolol have an additive effect, and carisoprodol, clonazepam, lorazepam, and zolpidem have a less-than-additive effect on locomotor response to nicotine.

Combination pharmacotherapy confers the advantage of targeting more than one molecular pathway while potentially reducing doses and minimizing aversive secondary effects. As varenicline has demonstrated superiority to bupropion for smoking cessation [29], we tested varenicline in combination with bupropion as well as each of the eight category (iv) medications. We used Bliss Independence, a mathematical model of drug interaction [30], to evaluate potential additive, synergistic, or antagonistic interactions. We co-administered each medication with varenicline at half the dose used singly. Varenicline and bupropion in combination showed a greater than expected attenuation yielding 66% of the untreated nicotine response compared to the theoretical 83% response assuming an additive model (**Fig. 5**). This suggests improved efficacy over monotherapy, consistent with human clinical trial data [31], [32]. Similarly, but to a lesser extent than bupropion, both topiramate (45% measured vs. 54% expected untreated nicotine response) and apomorphine (41% measured vs. 45% expected untreated nicotine response) demonstrated a greater-than-additive response with varenicline, suggesting candidate combination therapy strategies for evaluation in smoking cessation studies. Diazepam in combination with varenicline elicited an 87% measured nicotine response, nearly equivalent to the expected 86% nicotine response if the medications were acting in an additive manner. The remaining medications tested had a lesser-than-expected response in combination with varenicline. Betaxolol was close to maintaining efficacy, however, with a 46% measured versus 38% expected untreated nicotine response. Carisoprodol, zolpidem, clonazepam, and lorazepam failed to attenuate the nicotine response to the theoretical additive magnitude when co-administered with varenicline.

## Discussion

Repositioning of clinically available medications provides a strategy for addressing the expense and delay inherent in traditional drug development and the larval zebrafish model described here is a relevant and cost-effective tool for evaluating medications for repurposing as tobacco dependence treatments. Larval zebrafish as a preclinical model to study the biological effects of nicotine exposure has been established in recent years [11]. Our data recapitulates previously reported results using larval zebrafish to study dose-dependent, nicotine-induced locomotor activation [18], including the biphasic activation curve commonly observed for drugs of abuse [33]. The alpha and beta neural nAChR subunit encoding genes expressed in humans are conserved in the zebrafish. In addition, other receptor families and neurotransmitter pathways associated with drugs of abuse, addiction, and reward are also conserved between these species [12]. The high level of conservation in these key drug-response systems provide a strong genetic rationale for evaluating pharmacotherapeutics impacting a diverse set of neural pathways.

We comprehensively evaluated 39 FDA-approved medications vetted by a physician for the likelihood it would be prescribed to treat tobacco dependence based on side effects and contraindications if efficacy was found. We employed a CNS-mediated locomotor activation response assay that is readily attenuated following pretreatment with varenicline and bupropion, giving us the predictive clinical validity necessary to interpret our candidate medications as potential tobacco dependence treatment options. We tested 14 medications known to target the GABA system, 13 known to target other neural systems and 12 targeting non-neural systems (**Table S1**). Eight medications (apomorphine, betaxolol, carisoprodol, clonazepam, diazepam, lorazepam, topiramate, and zolpidem) from five drug classes attenuated the nicotine response without impacting the locomotor response to peripheral-acting stimuli (cinnamon and mustard oil). The medications shown to attenuate the acute nicotine response in this study have well described pharmacologic targets in dopaminergic, GABAergic, or adrenergic systems, which may explain their impact on nicotine response. Like most neural acting compounds, however, they may also impact other receptor systems in addition to their commonly associated targets. To determine the precise mechanisms by which these medications mediate their nicotine-attenuating effects requires further investigation.

**Figure 6:**
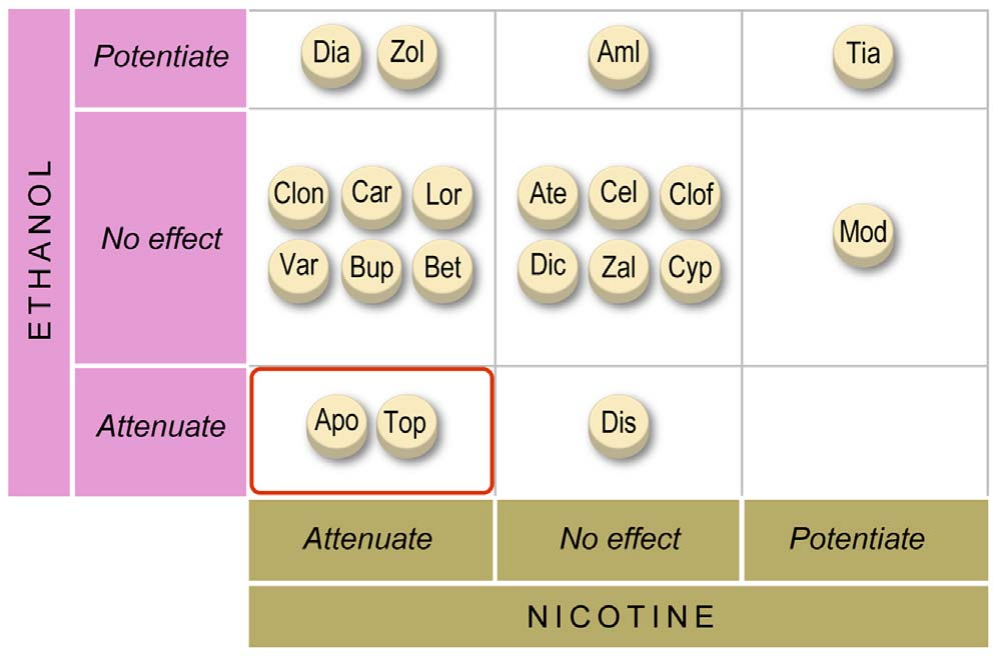
Distinct behavioral responses by FDA-approved medications on the nicotine and ethanol locomotor response. The ability to modulate the ethanol (Y-axis) or nicotine (X-axis) locomotor responses is shown. Apomorphine and topiramate attenuate both nicotine and ethanol responses.

To further characterize the specificity of response and better understand the mechanisms underlying the acute effects of nicotine and ethanol, nicotine-response modifiers and those medications showing evidence of absorption were evaluated for effects on ethanol locomotor response. Ethanol has a more diverse set of targets to elicit its rewarding effects [34], [35] compared to that of nicotine, and as such, is more difficult to validate in the same manner as the nicotine locomotor assay. We show that disulfiram blocks ethanol-induced locomotor activation, but disulfiram is a peripheral-acting ethanol-metabolism inhibitor, and therefore does not specifically block a CNS-mediated response. In the context of counter-screening compounds for tobacco dependence treatment, the ethanol assay allows us to assess general drug specificity with regard to blocking a locomotor response and may suggest impact on alcohol abuse treatment, but the results should be interpreted with this limitation in mind. Two medications attenuated both nicotine- and ethanol-induced locomotor response, one potentiated both responses (also potentiated cinnamon and mustard oil-induced locomotion), and the remaining 17 evaluated with ethanol specifically affected one stimulus or had no effect (**Fig. 6**). This suggests partially overlapping, yet largely unique pathways involved in the initiation of the locomotor response to nicotine and ethanol. The dual impact assessment of both nicotine and ethanol response suggests this locomotor assay system maintains specificity in identifying candidate compounds for drug repositioning and may be informative for the treatment of smokers with comorbid alcohol use disorders.

We further show combined treatment with varenicline and bupropion achieves an improved response over monotherapy, at half the dose, suggesting possible synergism in the modes of action. This improved efficacy over monotherapy is consistent with human clinical studies [31], [32]. Combination treatment of varenicline with either apomorphine or topiramate also revealed an improved attenuating response to nicotine, although at a lesser magnitude than that of varenicline with bupropion, suggesting potential benefit to their combined use to treat tobacco dependence. Developing alternative treatment strategies using lower doses of combined medications may alleviate the side effects of either monotherapy enabling the patient to continue treatment. Even if combination therapy does not improve efficacy, lessening aversive side effects while maintaining efficacy may allow patients previously not able to tolerate monotherapy doses to remain on efficacious pharmacotherapy. Of note, in addition to safety, toxicity, and contraindication information, drug interactions are known for many medications approved by the FDA, making translation of potential combination therapies into the clinic an informed process. In combination with varenicline, diazepam maintained efficacy, betaxolol was close to an additive response, while carisoprodol, zolpidem, and the other benzodiazepines showed a less-than-expected response. These medications may be less likely to find utility in combination therapy for smoking cessation or perhaps 50% of the efficacious dose is a sub threshold level to elicit a beneficial response.

Benzodiazepines, zolpidem and carisoprodol act on GABA(A) receptors to enhance GABA activity. Benzodiazepines are FDA approved as anticonvulsants, anxiolytics, and treatments for alcohol withdrawal, zolpidem is approved to treat insomnia, and carisoprodol is approved as a muscle relaxant. These medications are associated with varying degrees of abuse and dependence [36]–[38] providing important key constraints for use as therapeutic interventions for tobacco cessation. Carisoprodol also potentiates the effects of opioids making abuse and safety a concern in the use of this medication [39].

Betaxolol is a beta1-adrenergic antagonist approved to treat hypertension by the FDA. It inhibits cocaine-induced conditioned place preference at high doses [40], but not low doses [41], [42], and blocks opiate [43] and cocaine [44], [45] withdrawal-induced phenotypes. This suggests beta1-adrenergic receptors are involved in drug-induced phenotypes and may support our findings that betaxolol can attenuate nicotine-induced locomotor activation in larval zebrafish. Additionally, tobacco use is the most common preventable cause of cardiovascular disease [46], suggesting betaxolol, and other adrenergic antagonists, may have positive effects on comorbid hypertension and tobacco dependence.

Apomorphine, a non-specific D1 and D2 dopamine receptor agonist approved as an anti-Parkinsonism medication, has been extensively studied for dopamine receptor sensitivity and function [47], [48] and in treating alcohol dependence [49]–[51]. It modulates ethanol, morphine, and nicotine-associated behaviors [52]–[54]. In rats, apomorphine reverses nicotine-induced changes in the firing rates [55] and population activity [56] of dopamine neurons, and decreases nicotine self-administration [57]. Low doses of apomorphine stimulate presynaptic dopamine receptors to suppress cocaine-induced locomotion in rats [58], which is consistent with dopamine release being required for this response to a rewarding stimulus. This also suggests the use of apomorphine for the treatment of drug abuse may require smaller doses than those used to treat Parkinson's, a condition that requires the stimulation of the postsynaptic receptors using higher doses. The blunting effect of apomorphine on acute nicotine-induced locomotor response, in concert with it's ability to reverse neuroadaptations of the dopamine system following chronic nicotine exposure, makes apomorphine an appealing medication for treating tobacco dependence. Apomorphine has also been shown to decrease the locomotor-stimulating effects of ethanol [59], consistent with our results.

Topiramate is an FDA-approved anti-convulsant and treatment for migraines, and when paired with phentermine, obesity. Topiramate has been studied for treating alcohol dependence, showing efficacy over placebo for improving abstinence, decreasing craving and withdrawal symptoms, and improving quality of life measures [60]–[62]. Clinical studies have suggested efficacy in promoting smoking abstinence in alcohol-dependent smokers [60], [63], [64]. Our results showing topiramate attenuates nicotine and ethanol locomotor response in larval zebrafish are consistent with these studies.

The psychomotor activating theory of addiction describes the locomotor stimulating effect caused by drugs of abuse (including nicotine) as a corollary read-out of potential euphorigenic-like response [8]. While this does not represent the complete spectrum of phenomena associated with drug addiction, it does provide a component behavior model that is a rational path for evaluating pharmacotherapeutic blockade of nicotine's rewarding effects. The modulation of the locomotor response has been noted in other preclinical models [65]–[67] consistent with our observations in zebrafish.

Clinically relevant preclinical data has traditionally been derived from mammalian models. Emerging data from the zebrafish, a non-mammalian vertebrate, is providing new options for preclinical assessment, suggesting this model is appropriate and justified in certain contexts. Specifically, others and we believe that the use of zebrafish for the study of behavioral endophenotypes of psychiatric disorders and addiction can be uniquely advantageous [11], [13], [68]–[71]. In addition, an increasing number of zebrafish studies illustrate consistency with existing mammalian findings related to addiction and pharmacotherapeutic modulators of neurally mediated behaviors. Adult zebrafish assays have been developed to evaluate drug seeking/taking behaviors, impulsivity, withdrawal, and nicotine-induced changes in social interaction using nicotine-induced conditioned place preference [72], [73], 5-choice serial reaction time tests [74], anxiety measures [75], and shoaling experiments [76], respectively. The translational potential of many zebrafish behaviors as they relate to psychiatric disease have been reviewed elsewhere [7]. In addition, previous reports of medications used in our study show the zebrafish responses associated with the known drug actions to be consistent with mammalian data. Apomorphine induces biphasic locomotor responses [77] and modulates Parkinsonian phenotypes [78], and benzodiazepine anxiolytics block anxiety-related behaviors [79] in both zebrafish and mammalian models. This suggests the targets of these medications are conserved in zebrafish and are functioning equivalently.

The larval zebrafish locomotor activation assay provides a focused model unable to fully represent the complexities of tobacco or alcohol dependence or addiction in general and was not assessed for the potential of false positives with medications not found to be efficacious in people. While this assay is capable of identifying compounds blocking a neurologic reward response to nicotine exposure, it cannot identify compounds impacting other aspects of addiction, including withdrawal and contextual responses as well as other psychological or social factors influencing addiction-related behaviors. It may be informative to evaluate the candidate medications identified in this study in the more complex behavioral paradigms described above, or in mammalian model systems, but the imperative to first validate these assays with the current therapeutics for tobacco dependence for the results to be suggestive of predicting clinical efficacy would remain. Our perspective is that the ideal model system for therapy evaluation is the human, and human laboratories have been suggested for initial clinical screening [80]. Such a paradigm may be well suited for studying drug-repositioning candidates from preclinical animal model studies. The more connected and informed the bench to bedside research relationship is, the more likely appropriate and timely translation of findings may occur.

With known limitations in mind, we believe the data represented here indicates that the larval zebrafish model is a viable preclinical model to test pharmacological agents that may decrease the reward response to nicotine exposure in humans. This may, in turn, decrease the risk for relapse and increase tobacco abstinence rates. With the advent of the validated behavioral screen described here, primed with initial drug evaluation data, the development of a high-throughput screening method for pharmacotherapeutic modifiers of nicotine and ethanol response is now warranted, and a number of studies have shown measuring larval locomotion is amenable to high throughput approaches [69], [81], [82]. Moreover, exploiting this model to evaluate medications approved for human use by the FDA enables clinicians to study these medications in clinical trials without further preclinical safety testing.

## Supporting Information

Figure S1 **Larval behavior experimental setup.** (**A**) Image of cabinet, light plate, and camera with larvae in weighing dishes. (**B**) Close-up image of light plate with diffuser sheet, acrylic template, weighing dishes with larvae, and the penny.
(TIF)

Table S1 **Drugs studied in larval nicotine-induced locomotor activation assay.** FDA-approved indications and mechanism of action obtained from Micromedex.
(XLS)

Table S2 **Summary of drug evaluation.** All results are represented as the percent of stimulus only (untreated larvae) response averaged across three replicates (± SE); n = 30 larvae per condition. Ethanol (EtOH) experiments had six replicates; n = 60 larvae per condition. Nicotine, cinnamon oil, mustard oil and ethanol experiments were performed at 6 dpf with overnight pretreatment in the drug. For the 24-hour washout experiment (post wash nic), larvae were treated overnight in the drug, tested in the nicotine assay, rinsed of all drug, placed in fresh embryo water for 24 hours and retested with nicotine at 7 dpf. Acute early and acute late response represents the first 4 minutes and last 4 minutes (26–30 minutes), respectively, post drug exposure at 5 dpf. Students t-test for significance.
(XLS)
