# Supplementary figures and images for: Larval Zebrafish Model for FDA-Approved Drug Repositioning for Tobacco Dependence Treatment
Source: PLoS One. 2014 Mar 21;9(3):e90467. doi: 10.1371/journal.pone.0090467 (PMC3962344; doi:10.1371/journal.pone.0090467)

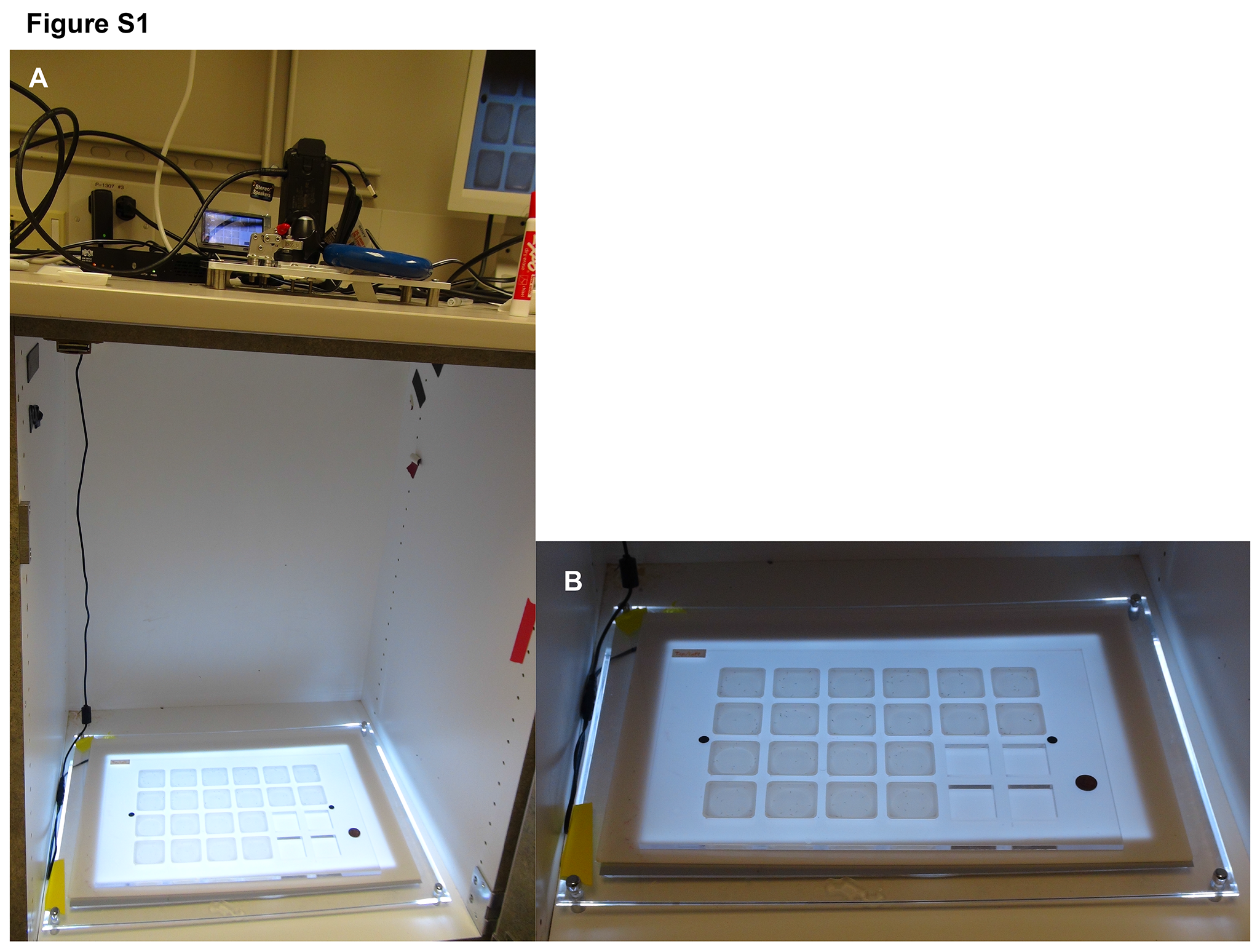

Supplement: Figure S1 — Larval behavior experimental setup. (A) Image of cabinet, light plate, and camera with larvae in weighing dishes. (B) Close-up image of light plate with diffuser sheet, acrylic template, weighing dishes with larvae, and the penny. (TIF) [file pone.0090467.s001.tif]
